# Recent Progress in Photoresponsive Biomaterials

**DOI:** 10.3390/molecules28093712

**Published:** 2023-04-25

**Authors:** Miriam Di Martino, Lucia Sessa, Rosita Diana, Stefano Piotto, Simona Concilio

**Affiliations:** 1 Department of Pharmacy, University of Salerno, Via Giovanni Paolo II 132, 84084 Fisciano, Italy; midimartino@unisa.it (M.D.M.); lucsessa@unisa.it (L.S.); piotto@unisa.it (S.P.); 2 Bionam Research Centre for Biomaterials, University of Salerno, Via Giovanni Paolo II 132, 84084 Fisciano, Italy; 3 Department of Agricultural Sciences, University of Naples Federico II, Via Università 100, 80055 Portici, Italy; rosita.diana@unina.it

**Keywords:** photoresponsive, azobenzene, hydrazones, diarylethene, spiropyran, biomaterials

## Abstract

Photoresponsive biomaterials have garnered increasing attention recently due to their ability to dynamically regulate biological interactions and cellular behaviors in response to light. This review provides an overview of recent advances in the design, synthesis, and applications of photoresponsive biomaterials, including photochromic molecules, photocleavable linkers, and photoreactive polymers. We highlight the various approaches used to control the photoresponsive behavior of these materials, including modulation of light intensity, wavelength, and duration. Additionally, we discuss the applications of photoresponsive biomaterials in various fields, including drug delivery, tissue engineering, biosensing, and optical storage. A selection of significant cutting-edge articles collected in recent years has been discussed based on the structural pattern and light-responsive performance, focusing mainly on the photoactivity of azobenzene, hydrazone, diarylethenes, and spiropyrans, and the design of smart materials as the most targeted and desirable application. Overall, this review highlights the potential of photoresponsive biomaterials to enable spatiotemporal control of biological processes and opens up exciting opportunities for developing advanced biomaterials with enhanced functionality.

## 1. Introduction

**Figure 1:**
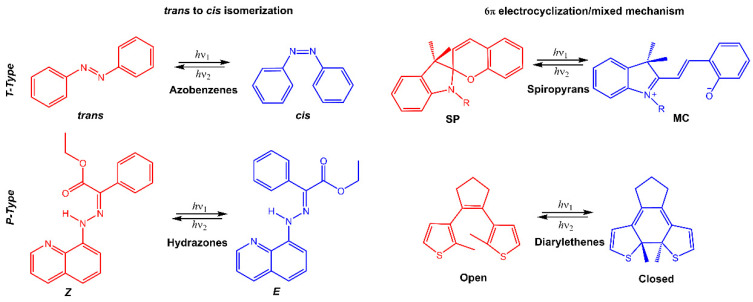
Chemical structures of the most used photoresponsive molecules.

Stimuli-responsive molecules, which an external stimulus can manipulate, have recently received considerable interest. The use of light as a stimulus is fascinating for its precise spatiotemporal control and non-destructive nature, as the intensity and wavelength can be easily regulated [1,2,3]. Light-responsive molecules, also known as photoresponsive molecules, are a class of compounds that undergo reversible or irreversible structural changes upon exposure to light. The changes in their structure can alter their physical or chemical properties, such as solubility, polarity, or reactivity. This ability to respond to light has made light-responsive molecules an attractive area of research in many fields, including chemistry, materials science, and biology [4]. When chromophores are irradiated with light of a specific wavelength, they can undergo a reversible or irreversible conversion between isomeric forms or ring-opening/closure. One of the most studied types of light-responsive molecules is azobenzene. Azobenzene has a unique structure consisting of two benzene rings connected by a central nitrogen–nitrogen bond. When exposed to UV light, azobenzene undergoes a reversible *trans*-*cis* isomerization, changing from a linear to a bent conformation. The *cis* form is stable without light, but upon exposure to visible light or heat, it reverts to the *trans* form. This reversible photoisomerization has led to the development of many applications, such as molecular switches, sensors, and photoresponsive materials. Among photoswitches, azobenzenes, and their heteroaromatic analogs, like stilbenes and hydrazones, undergo double-bond isomerization. Diarylethenes undergo cyclization; spiropyrans follow a mixed mechanism, as they experience electrocyclization first and then *trans-cis* isomerization (Figure 1) [5,6,7]. Spiropyran has a unique structure consisting of two rings connected by a single carbon atom. The two rings are typically a benzene ring and a pyran ring, giving the molecule a bicyclic structure. The spiropyran group is sensitive to UV light; it can be converted from its closed, non-polar form (spiropyran, SP) to its open, polar form (merocyanine, MC) when exposed to the light of a certain wavelength (Figure 1). This ring-opening reaction is reversible and can be triggered by visible light or heat, leading to the development of photochromic materials and sensors. Diarylethene molecules are compounds that have been discovered relatively recently, and they possess a distinctive characteristic of transitioning from colorless to red when exposed to light. Diarylethene compounds provide several advantages, such as excellent thermal stability and high quantum yield for both their isomers. Due to these properties, they are well-suited for application in photoresponsive materials, exhibiting robust performance.

Many of these molecules exhibit positive photochromism (P-type), whereby the photochemically induced species has an absorption maximum λ_max_ (metastable) that is shifted to longer wavelengths (bathochromic shift) compared to λ_max_ (stable). However, if the initial photoisomer undergoes a decolorization (bleaching) during photoisomerization, and λ_max_ (stable) is greater than λ_max_ (metastable), then the molecule displays negative or inverse photochromism (T-type) [8]. When inserted into a polymeric matrix, the conversion of photochromic molecules modifies the properties of the polymer, such as polarity, hydrophilicity, solubility, or electrical and optical properties.

Photoresponsive biomaterials are created by integrating photoresponsive molecules, such as spiropyrans, azobenzenes, hydrazones, and diarylethenes, into biomaterials like hydrogels, nanoparticles, or scaffolds. This development has the potential to revolutionize biomedicine by enabling new approaches to drug delivery, tissue engineering, and imaging. These materials can be controlled and manipulated using light, making them an increasingly prominent part of biomedical research in fields like tissue engineering, dynamic hydrogels for cell encapsulation, controlled drug delivery, biosensors, and microdevices [9,10,11]. Additionally, photoresponsive biomaterials are versatile platforms for various biomedical applications because they can be engineered and designed with specific properties and functionalities.

This review aims to present recent years’ innovation on photoresponsive molecules, with particular attention to the four classes of compounds: azobenzene, hydrazones, diarylethene, and spiropyran. For each category of photoswitches, applications in materials such as hydrogels, films and coatings, and metal-organic frameworks (MOFs) are explored in detail.

## 2. Azobenzene Photoswitches

**Figure 2:**
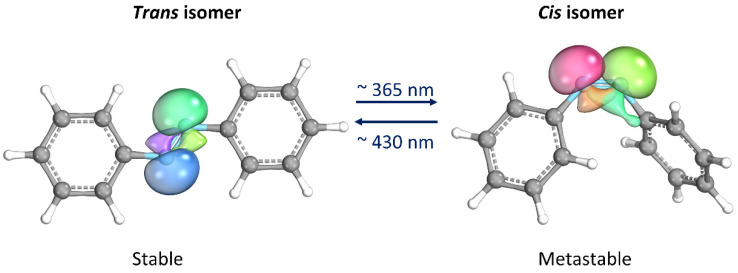
Photoisomerization process of azobenzene. Lone electron pairs on the nitrogen atoms are evidenced. This image has been realized with the IboView program [17].

Thanks to its simple molecular structure and unique characteristics, azobenzene (AB) has probably been the most studied photoswitches since its reversible isomerization was first reported about eight decades ago [12]. When the *trans* isomer, which is thermodynamically stable, is exposed to UV light (λ ≈ 365 nm), it isomerizes into the *cis* form. The latter isomer is metastable, i.e., spontaneously returning to the *trans* form once the UV source is removed. The return to the *trans* form can be significantly accelerated by heating or exposure to visible light (λ ≈ 430 nm) [13]. Moreover, they proceed with high quantum yields and are completely reversible. The most important aspect is that the two isomers differ significantly in their geometries and electronic properties, making AB an attractive component for the design of photoswitchable materials [14,15]. Some materials containing AB can convert light into mechanical energy due to the change in molecular geometry that occurs during AB isomerization. Additionally, the molecule’s polarity undergoes a significant change as lone electron pairs on the nitrogen atoms take on opposite orientations in the *trans* configuration, resulting in a non-polar molecule. On the other hand, in the *cis* isomer, the lone electron pairs arrange themselves in a way that increases the molecule’s dipole moment, typically ranging from 3.1–4.4 D compared to 0–1.2 D in the *trans* configuration (Figure 2) [14,16].

Several studies have reported the synthesis of azobenzenes and their derivatives for potential use in biomedical applications due to their various biological properties, ranging from antioxidant, antiviral, and antimicrobial to antitumor and antidiabetic [13,18,19,20,21]. Introducing these molecules into polymer matrices has resulted in the development of intelligent biomaterials, known as ‘smart’ materials. These materials can respond to light energy and cause large-scale changes or can utilize isomerization to provide intrinsic activities, such as antibacterial properties [22].

### 2.1. Azobenzene-Based Hydrogels and Particles

**Figure 3:**
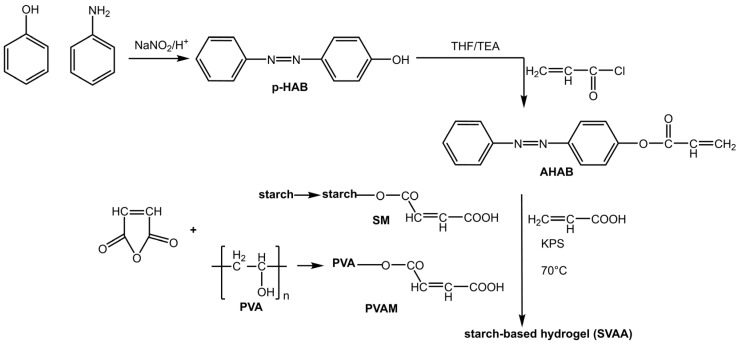
Preparation of starch-based hydrogel containing azo and carboxyl groups [24].

An interesting area of application for azobenzene as a photo-commutator is photoresponsive hydrogels: soft, flexible, hydrophilic polymers that swell in water. Hydrogels have attracted particular attention for their potential biomedical applications, such as self-healing, soft tissue engineering, drug delivery, and wound healing [23]. However, their weak mechanical strength significantly hinders their performance. The practical and reversible photoisomerization of azobenzene allows for the regulation of the mechanical properties of the gel. Su et al. [24] prepared a starch-based hydrogel with an incorporated AB based on polyvinyl alcohol and acrylic acid (Figure 3):

This hydrogel showed a dual response: a photoresponse due to the presence of azobenzene groups (4-hydroxy azobenzene (*p*-HAB)) and a pH response due to the starch-based macromonomers and polyvinyl alcohol (PVA). 

**Figure 4:**
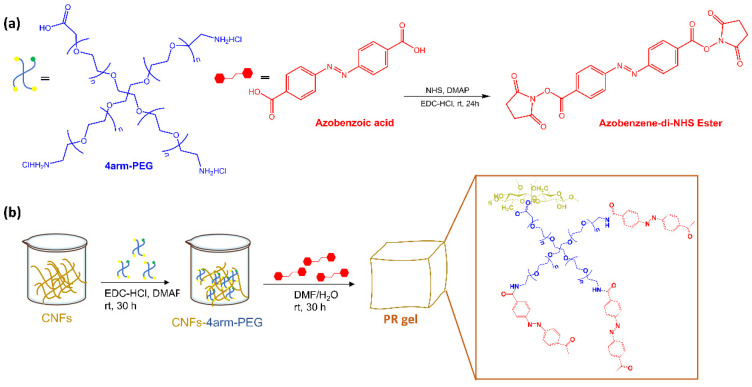
(**a**) Structures of polymers 4arm-PEG and the synthetic procedure of Azo-NHS and (**b**) Schematic illustration of the preparation procedures of PR-gel [25].

L. Dai et al. [25] reported a new photoresponsive hydrogel (PR-gel) (Figure 4) developed by integrating 4arm-polyethylene glycol (PEG) and azobenzene, as photoswitches, into cellulose nanofibrils (CNFs). 

**Figure 5:**
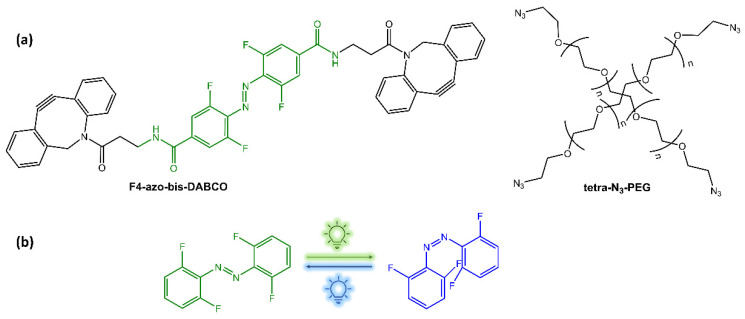
(**a**) Building blocks of photochromic hydrogels; (**b**) isomerization under green and blue light-emitting diodes (LEDs) irradiation [26].

Under UV irradiation, the photoisomerization of azobenzene in the PR-gel caused softening of the hydrogel, allowing the photocontrolled release of bovine serum albumin. The hydrogel also exhibited good mechanical strength, stability, and reversible structure recovery, as well as biocompatibility. Zhao et al. [26] have functionalized a PEG hydrogel with orthofluoroazobenzene as a crosslinker. Upon irradiation with blue and green light, this hydrogel showed a reversible photo-modulation of its elasticity (Figure 5). 

This system avoided UV light irradiation, which affects the photostability of materials and can also be a problem for their use in biological applications. More recently, a new class of hydrogel, supramolecular hydrogels, has been developed. These are formed by the self-aggregation of low molecular weight compounds through a combination of non-covalent interactions. Since these are weak interactions, they can be easily broken when exposed to light. Among the possible non-covalent interactions, host-guest interaction is an important one. Thanks to their low toxicity, the most used guest molecules are cyclodextrins (β-CD). When the azobenzene is in the *trans* configuration, it enters the cyclodextrin cavity. Following UV irradiation, when the azobenzene is in the *cis* configuration, it exits from the cavity [27]. 

Y. Kim et al. [28] introduced multifunctional gels that contained a host-guest complex between azobenzene-grafted carboxymethyl cellulose (CMC-Azo) and β-cyclodextrin, linked via disulfide bonds with agarose.

**Figure 6:**
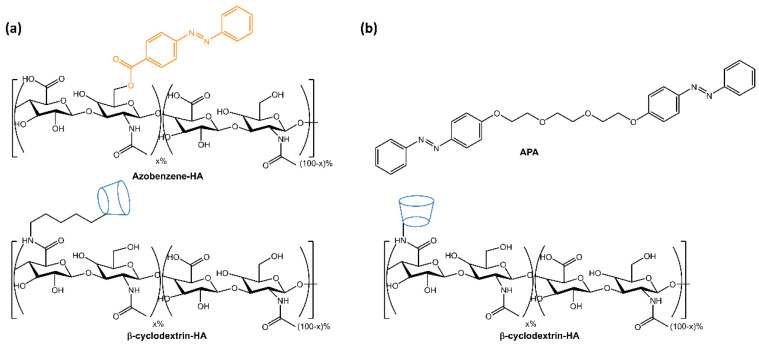
Chemical structures of supramolecular photoresponsive hyaluronic acid-based hydrogels by (**a**) Rosales et al. [29] and (**b**) Gao et al. [30].

The resulting hydrogels showed self-healing properties through host-guest complexation and gel-sol transition following UV light-induced azobenzene isomerization. The self-regeneration capacity was confirmed through tensile tests, and drug release was accelerated by 80% within 3 h using UV light. The hydrogels were non-cytotoxic and had the potential as biomedical materials for the development of drug delivery systems. Rosales et al. [29] reported the design of hyaluronic acid-based hydrogels that used light to reversibly modulate the hydrogel properties through supramolecular crosslinking formed by azobenzene linked to β-cyclodextrin (Figure 6a). The mechanical properties of the hydrogel and network connectivity could be modified by altering the binding affinity between azobenzene and β-cyclodextrin using different wavelengths of light. The hydrogels had potential applications in drug delivery and mechanobiology, as they allow for temporal regulation of the material properties. In the same way, in a recent paper by Gao et al. [30], a hyaluronic acid-based supramolecular gel was formed using an amphiphilic azobenzene (APA) and β-cyclodextrin (Figure 6b). The hydrogels exhibited a photo- and thermo-responsive sol-gel transition due to host-guest interactions between Azo and β-CD and intermolecular hydrogen bonds of hyaluronic acid. 

**Figure 7:**
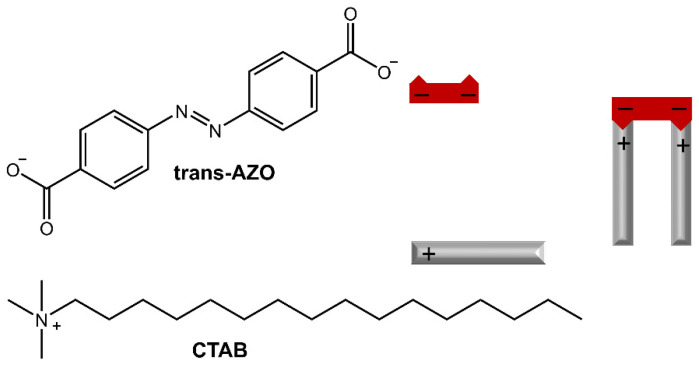
Chemical structures and schematic of the self-assembly of CTAB and AZO molecules [31].

Furthermore, the release of Rhodamine B from the hydrogel could be controlled by irradiation with UV light or high temperature. In the work of Salzano de Luna et al. [31], the design of a supramolecular hydrogel formed by self-assembly between azobenzene-4,4′-dicarboxylic acid (AZO) and cetyltrimethylammonium bromide (CTAB) in a molar ratio of 2:1 CTAB: AZO was reported (Figure 7). 

**Figure 8:**
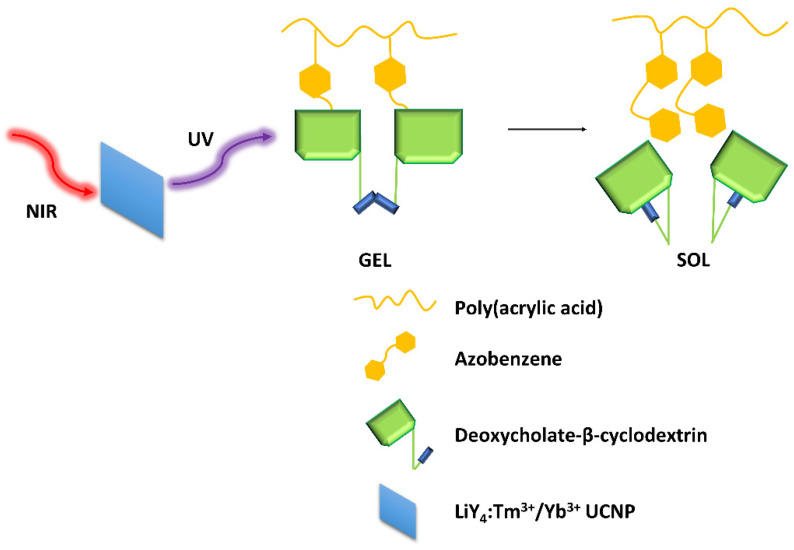
Graphical demonstration of the interactions within the hydrogel matrix [32].

The hydrogels showed self-healing capabilities when left at rest after stress-induced damage. The AZO isomerization induced by light also gave the gel a light-responsive property. To avoid using a UV light for excitation, Mandl et al. [32] modified a supramolecular hydrogel composed of an azobenzene-modified poly(acrylic acid) copolymer and deoxycholate-β-cyclodextrin with lanthanide-doped nanoparticles (LiYF_4_:Tm^3+^/Yb^3+^) (Figure 8), which emitted UV light upon NIR excitation. A complete gel-sol transition was observed within 60 min with irradiation at 980 nm.

**Figure 9:**
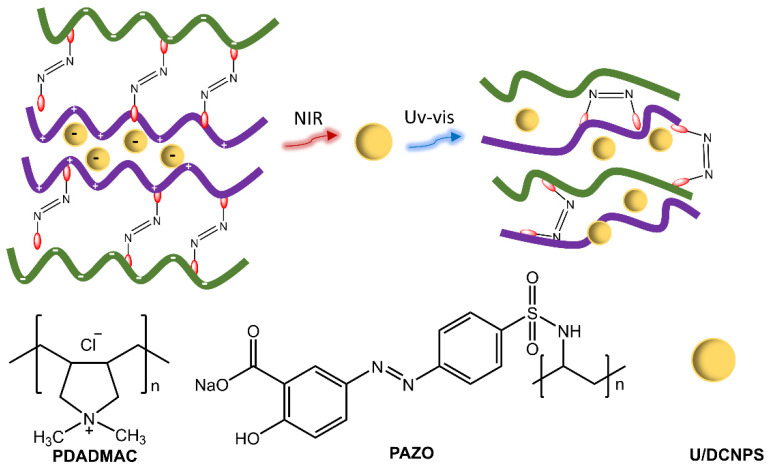
Illustration of up/down conversion nanoparticle (U/DCNP) functionalized hollow polymer nanocapsules and the near-infrared light-induced decomposing process from 180 nm nanocapsules to scattered polymers and 20 nm U/DCNPs by [35].

Polymer particles with sizes ranging from nano to micrometer have practical uses in various fields, such as adhesives, cosmetics, inks, and paints, as well as potential applications in the environmental, optical, electrical, and medical areas. Polymer particles with well-designed surfaces have potential applications in drug delivery. Photoresponsive polymer particles that change in response to light enable the regulation of drug delivery [33,34]. Zhao et al. [35] showed the development of nanocapsules consisting of polymers functionalized with azobenzene groups and up/down conversion nanoparticles (U/DCNPs), which emitted in the UV/visible region following excitation in the NIR, triggering the photoisomerization of the azobenzene groups in the polymer structure (Figure 9). As a result, the nanocapsules could decompose and change from large initial sizes (approx. 180 nm) to small U/DCNPs (approx. 20 nm). The polymeric species, poly(diallyl dimethylammonium chloride) (PDADMAC) and poly[1-(4-(3-carboxy-4-hydroxy-phenyl azo) benzenesulfonamide)-1,2-ethanediyl] (PAZO) with positive and negative charges, respectively, were assembled layer by layer through electrostatic interaction on the surface of colloidal SiO_2_ nanoparticles. The composites were anchored with negative charges to U/DCNPs.

The advantages of these nanocapsules were their ability to avoid biological barriers and ensure a prolonged circulation time in the blood. They could also accumulate in cancers four times more effectively than in normal tissues and, after NIR-induced dissociation, could be rapidly eliminated from cancers within one hour and release loaded drugs for chemotherapy. In recent work, Zhou et al. [36] used upconverting nanoparticles coated with photoswitchable azobenzene and cyclodextrin and loaded with microglia activator, bacterial lipopolysaccharide (LPS). NIR light-induced photoisomerization of the Azo group and subsequent dissociation of β-CD produced the release of LPS.

### 2.2. Azobenzene-Based Films and Coatings

**Figure 10:**
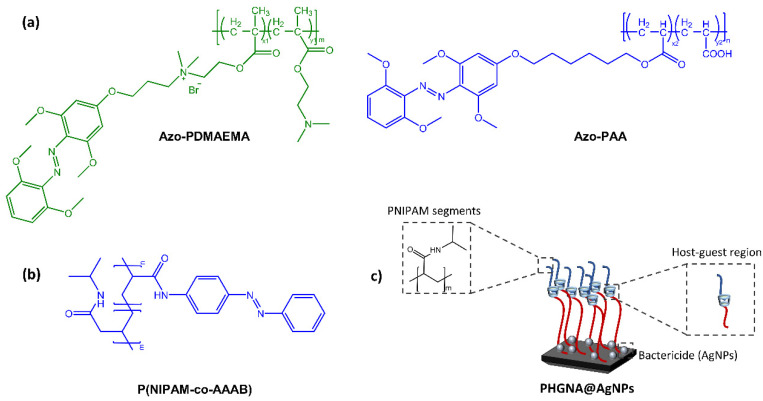
(**a**) Chemical structures of azo-functionalized polymers [37], (**b**) Chemical structures of azo-functionalized PNIPAM segment, and (**c**) Schematic of P(HEMA-co-GMA) brush polymeric surface to realize their multiple functions [38].

When azobenzene is incorporated into a polymer matrix to form a thin film or coating, it can exhibit interesting and functional properties. For example, the photoisomerization of the azobenzene can lead to changes in the surface topography and wettability of the coating, which can be exploited in various applications such as optical data storage, photoresponsive adhesives, and responsive membranes. Wang’s research group [37] synthesized a light-reactive surface with switchable adhesive and antibacterial properties. The azo-functionalized polymers, poly(6-((2,6-methoxyphenyl)azo-4-(2′,6′-dimethoxy)phenoxy)propyl dimethylaminoethyl methacrylate-coDMAEMA) (mAzo-PDMAEMA) and poly(6-((2,6-dimethoxy phenyl) azo-4-(2,6-dimethoxy) phenoxy)hexyl acrylate-co-acrylic acid) (mAzo-PAA) exhibited antimicrobial and bioadhesive properties respectively, while the substrate was β-CD-grafted silica (Figure 10a).

Light irradiation, resulting in the isomerization of the azobenzene moiety, promotes or hinders surface coating via host-guest interaction between azobenzene and cyclodextrin. In the field of surfaces with antibacterial properties with potential biomedical and surgical applications, Ni et al. [38] have developed an intelligent triple-function surface (Figure 10b,c) by integrating temperature-reactive poly(*N*-isopropyl acrylamide) (polyNIPAM), photoreactive Azo/CD complex, hydrophilic segments of poly(2-hydroxyethyl methacrylate) (poly HEMA).

Specifically, the hydration layer generated by the hydrophilic segments of poly(2-hydroxyethyl methacrylate) (PHEMA) prevented bacterial adhesion and subsequent proliferation. In contrast, the synergistic effect of the poly(*N*-isopropylacrylamide) (PNIPAM) chain and the dissociation of the host-guest azobenzene/cyclodextrin (Azo/CD) complex significantly promoted the release of bacteria in response to temperature change and UV light. Thus, the resulting surface simultaneously displayed three successive antimicrobial functions: it resisted ∼84.9% of the initial bacterial attack, killed 93.2% of the inevitably adhered bacteria, and released over 94.9% of the killed bacteria even after three cycles. Based on the same concept is the surface recently developed by Zheng et al. [39]. The material consisted of an azo-functionalized antibacterial and antifouling polymer, poly((2-(methacryloxy)ethyl)) trimethyl ammonium chloride (Azo-PMETAC), and an azo-modified polymer (sulfobetaine methacrylate) (Azo-PSBMA). The two polymers were anchored to a poly(2-hydroxyethyl methacrylate) (PHEMA) surface containing cyclodextrin groups.

**Figure 11:**
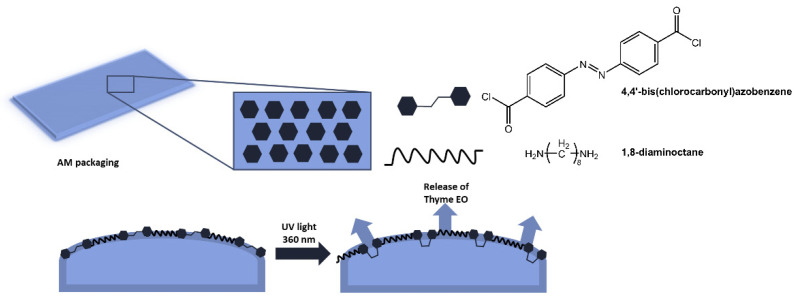
Antimicrobial active packaging based on polyethylene and polylactic acid films functionalized with photoreactive nanocapsules with thyme essential oil [42].

The authors demonstrated that the new material, with excellent antibacterial properties, not only inhibited bacterial growth, but upon isomerization of the azobenzene units, the polymer structure broke down, and all bacteria on the surface were released. Furthermore, following irradiation with visible light at 450 nm, the surface could regain antibacterial properties. Antimicrobial polymer films are also used in food packaging to reduce waste and increase the quality standards of perishable products [40,41]. In a paper by Marturano et al. [42], the use of photoreactive nanocapsules containing thyme essential oil as functional coatings for polyethylene and polylactic acid films to obtain active antimicrobial packaging was reported (Figure 11). The role of the azobenzene molecule was to promote the release of the active ingredient.

The effectiveness of the light-triggered release system was confirmed by the eight-fold increase in the concentration of thyme oil in the headspace of the films after UV exposure compared to non-irradiated films.

### 2.3. Azobenzene-Based MOFs

**Figure 12:**
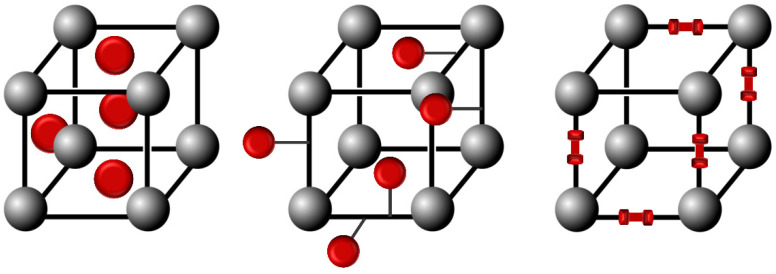
Schematic representation of the integration of azobenzene molecules into MOFs structures.

Azobenzene units are usually integrated into metal-organic-frameworks (MOFs) structures as pore-filling guests, pendant groups, or scaffold backbone incorporation to obtain the photoswitchable azobenzene-containing MOFs, as represented in Figure 12 [43,44].

**Figure 13:**
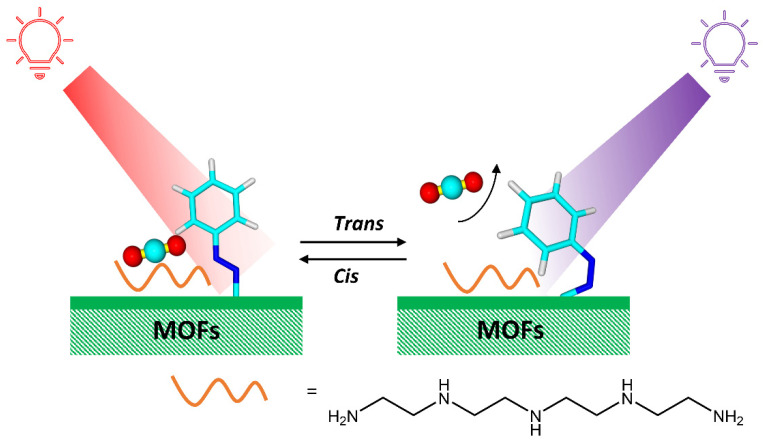
Tailored CO_2_ capture on photoresponsive MOFs through an interaction between active sites and photoresponsive molecules driven by photoisomerization [47].

Photoswitchable azobenzene-containing MOFs have potential applications in the separation of gas or liquid mixtures due to light-induced adjustment of the pore size [45]. Prasetya et al. [46] have synthesized a new generation-2 light-responsive MOF, Azo-MOF-1, with an azobenzene photoresponsive compound. The presence of the azobenzene group in Azo-DMOF-1 was also beneficial in making the MOF photoresponsive toward CO_2_, which was applicable for low-energy CO_2_ capture and, also, CO_2_/N_2_ selectivity. At the same time, Jiang et al. [47] have developed photoresponsive metal-organic frameworks (PMOFs) for CO_2_ adsorption by introducing tetraethylene pentamine into azobenzene-functionalized MOFs (Figure 13).

Amines are specific active sites for CO_2_ and help to capture CO_2_ selectively. The isomerization of azobenzene triggered by UV/Vis light significantly regulates the electrostatic potential of amines, modulating the adsorption of CO_2_ on strong, active sites.

In recent work, Chen et al. [48] have developed a novel photoswitchable metal-organic framework (MOF) thin film (Cu_2_(AzoBPDC)_2_ with azobenzene photoswitches side groups to realize the reversible remote-controlled switching.

Thanks to the reversible photoisomerization of the azobenzene moiety under UV (365 nm) and Visible (450 nm) irradiation, the authors have obtained the remote-control mode for the diffusion flow of polar gas molecules in MOF thin film. In fact, when the azobenzene switched to the *cis* state, the diffusion flux of the polar molecules significantly increased, but that of the non-polar molecules did not change. In another work [49], the diffusion behaviors of CO_2_ and N_2_ gases on a series of MOFs systems based on Mg-MOF-74-III as a platform and a chain containing arylazopyrazole, modified with methylene amine as the functional molecule have been investigated. The diffusion of CO_2_ was regulated by *cis*-to-*trans* isomerization of the functional unit.

**Figure 14:**
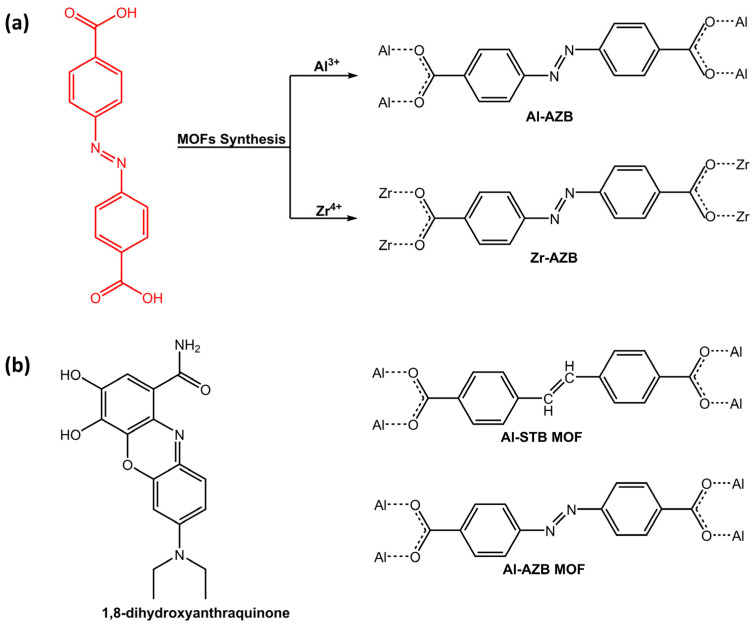
Chemical structure of (**a**) azobenzene dicarboxylic acid ligand and its respective MOFs (Al-AZB and Zr-AZB) by Mogale et al. [50]; (**b**) 1,8-dihydroxyanthraquinone, azobenzene (Al-AZB) and stilbene-based (Al-STB) MOFs [51,52].

Another application of photoresponsive azo-based MOFs was dye adsorption. In a work of Mogale et al. [50], two microporous photoresponsive azobenzene dicarboxylate MOFs of Al^3+^ (Al-AZB) and Zr^4+^ (Zr-AZB) (Figure 14a) were synthesized for the adsorption of Congo red (CR) dye. The Al-AZB demonstrated more effective adsorption of CR in comparison to the Zr-AZB due to much higher surface area, pore volume, and pore size. Conversely, Zr-AZB exhibited a faster dye removal. The results demonstrated the efficacy of synthesized photoreactive MOFs as highly capable materials for dye adsorption. For the same purpose, Parsa et al. [51] have developed a fluorescence sensor, 2D-metal-organic framework (TMU-54), containing the azobenzene group for the determination of quinone, 1,8-dihydroxyanthraquinone (danthron) (Figure 14b).

Investigations revealed that Lewis basicity and electron donation of azobenzene significantly affected danthron sensing by TMU-54. Aluminum-based MOFs containing azobenzene and stilbene dicarboxylic acid as linkers were studied by Ernst H.G. Langner’s research group [52] in recent work for celestine blue dye adsorption. Both azobenzene-based MOF (Al-AZB) and stilbene dicarboxylic acid-based MOF (Al-STB) (Figure 14b) exhibited favorable characteristics that make it efficient in adsorbing celestine blue from a solution, thanks to hydrogen bonding interaction. While azobenzene linker displayed *trans*-*cis* photoisomerization when exposed to UV and ambient light, stilbene linker did not show photoisomerization and only the *trans* isomer was observed, even after prolonged exposure to UV irradiation at 365 nm.

## 3. Hydrazone Photoswitches

**Figure 15:**
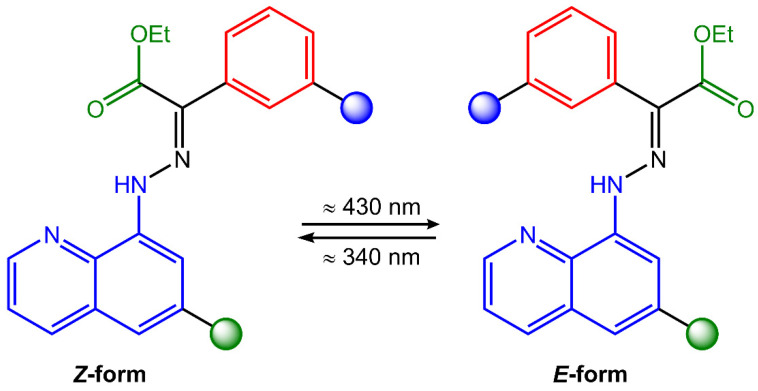
Chemical structure and photoisomerization process of hydrazone molecule.

Hydrazones are organic compounds that contain a carbon–nitrogen double bond (C=N) that links a hydrazine functional group (-NHNH_2_). They can be considered derivatives of hydrazine (H_2_NNH_2_) in which one of the hydrogen atoms has been replaced by a carbonyl group (C=O). Hydrazones can be synthesized by the condensation reaction of a hydrazine derivative with a carbonyl compound such as an aldehyde or a ketone. This reaction is typically catalyzed by an acid catalyst, such as hydrochloric acid or sulfuric acid [53]. Hydrazones have a wide range of applications, including as intermediates in the synthesis of pharmaceuticals, as ligands in coordination chemistry, and as chromogenic and fluorogenic sensors for various analytes [54]. They also exhibit biological activities, such as antitumor, antimicrobial, and antiviral properties. Hydrazones can undergo *trans*-to-*cis* isomerization of the C=N bond upon light exposure (Figure 15).

Hydrazones also undergo photochromism, the reversible change of color or absorbance upon irradiation with light. The photochromic behavior of hydrazones is typically attributed to the formation of a tautomeric form upon photoisomerization, which has a different absorption spectrum than the original form. The tautomeric form can then revert to the original state upon removal of the light source. As a result, hydrazone photoswitches have been adopted in various adaptive materials, such as liquid crystals, hydrogels, actuating polymers, and nanoparticles for drug release [55].

### 3.1. Hydrazone-Based Hydrogels and Particles

One example of a photoresponsive hydrazone biomaterial is a hydrogel that can undergo reversible changes in its mechanical properties upon exposure to light. This hydrogel is made by crosslinking a hydrazone-containing polymer with a light-sensitive crosslinker, which can be activated by UV light. When the hydrogel is exposed to UV light, the crosslinker forms additional crosslinks within the polymer network, leading to a stiffer and more stable hydrogel. However, when the light is removed, the crosslinks break down, and the hydrogel returns to its original, softer state.

**Figure 16:**
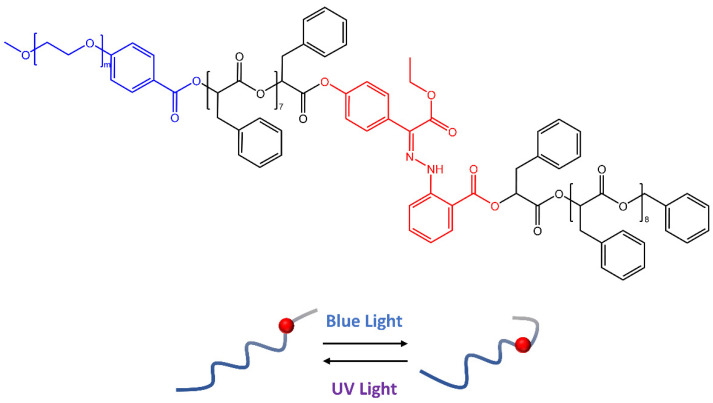
Schematic representation of the reversible shape transformation of self-assembled structures of PEG-b-[OPLA] containing hydrazone-based photoswitches via configurational switching of the system [56].

In a recent work by Wang [56], hydrazone molecules have been used to create polymeric vesicles capable of releasing cargo molecules on demand by responding to specific stimuli. The synthesis of amphiphilic block copolymers based on oligo(phenylactic acid) (OPLA) monodispersed molecular weight, with hydrazone photoswitches at particular locations, was reported in the paper. Upon light irradiation, the OPLA block (Figure 16) changed its conformation thanks to hydrazone photoswitches *E*-*Z* isomerization. In addition, the position and number of hydrazonic photoswitches in the monodisperse OPLA block were important factors for the reversible shape transformation of the polymer vesicles from an isotropic to an anisotropic morphology.

**Figure 17:**
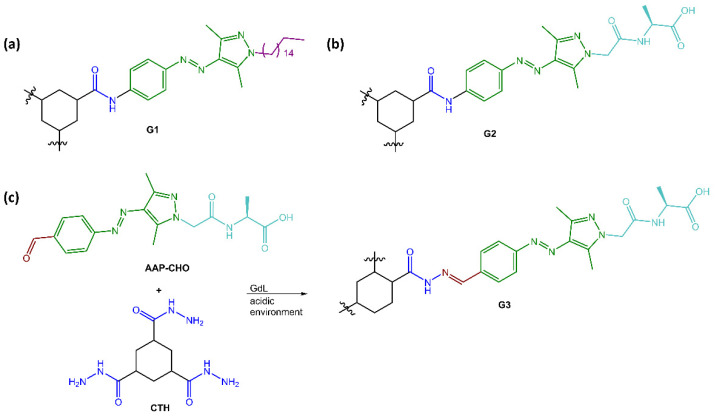
Chemical structures of (**a**) organogelator G1, (**b**) hydrogelator G2 and (**c**) reaction scheme of hydrazone-linked hydrogelator G3 [57].

Ravoo and co-workers [57] have developed versatile photoresponsive gels using tripodal low molecular weight gelators (LMWGs) with a cyclohexane-1,3,5-tricarboxamide (CTA) core that induced self-assembly of supramolecular polymers through face-to-face hydrogen bonding and planar conformation. The CTA core was substituted with three arylazopyrazole (AAP) arms to make the gels photoresponsive and coupled AAP to the core through hydrazones, allowing the hydrogelator and the resulting photoresponsive hydrogel to be assembled and disassembled using dynamic covalent chemistry (see Figure 17).

The researchers employed UV and visible light irradiation to control the stiffness of the hydrogels. They observed that the *E* conformation of the hydrogelators, which was more stable and planar, exhibited a higher storage modulus. In contrast, the *Z* isomer of both G2 and G3 caused a disruption of the planarity and the additional π-π interactions provided by AAPs, leading to a lower storage modulus and a softer gel.

**Figure 18:**
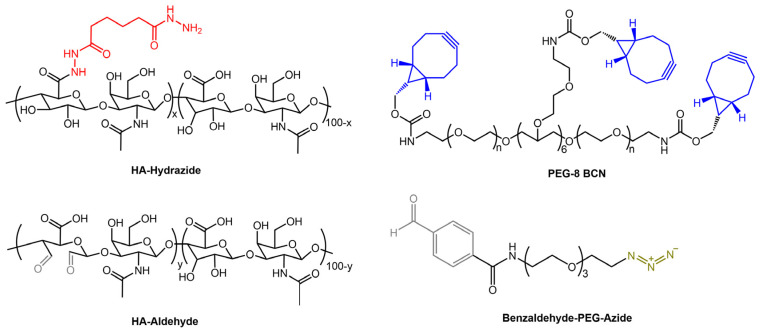
Chemical structures of hydrogel components: HA-Hydrazide, HA-Aldehyde, PEG-8-BCN, and Benzaldehyde-PEG-Azide [58].

Recently, Borelli et al. [58] have developed stress-relaxing hydrogels that combine biopolymer and synthetic macromer components to form hybrid networks. The hyaluronic acid was functionalized with an aldehyde or hydrazide groups to produce covalent hydrazone networks. Then, the authors used poly(ethylene glycol) functionalized with bicyclononyne and heterobifunctional small molecule crosslinkers that contained azide and benzaldehyde moieties (Figure 18) for stabilizing covalent networks. By adjusting the composition of the gels, the researchers could control the characteristic timescale for stress relaxation and the degree of stress relaxation that occurred.

The authors aimed to develop a photoresponsive hydrogel with tunable properties for the expansion and release of MSCs, mesenchymal stem/stromal cells, used in cell therapies.

**Figure 19:**
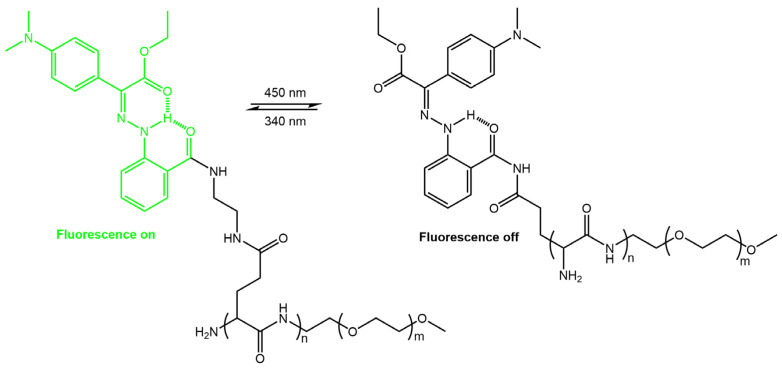
Schematic illustration of the switchable hydrazone-based molecule upon 450 nm irradiation, leading to the disruption of the nanoparticles and subsequent triggering of drug release [59].

Guo et al. [59] have developed a photochromic nanoparticle system (LSNP) that could be used to monitor their localization in tumor cells to improve the precision of controlled drug release, thus enhancing the efficiency of drug delivery. Their system was formed by assembling amphiphilic copolymers modified with fluorescent bistable hydrazone photoswitches (Figure 19).

**Figure 20:**
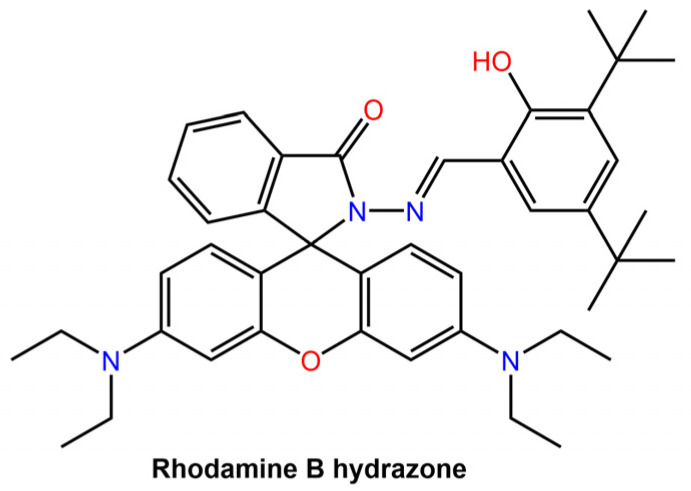
Chemical structure of Rhodamine B hydrazone used by Khosravi et al. for immunomagnetic particles [60].

The intrinsic emission of the hydrazone switch allowed for the observation of the particles being taken up by cells and their subsequent distribution within the cells. Upon *Z* to *E* photoswitching of the hydrazone switch within the nanoparticles, the particles expanded, leading to drug release and accompanied by emission quenching. The degree of quenching was used as an internal indicator to determine the amount of drug released. Khosravi et al. [60] reported the fabrication of immunomagnetic nanoparticles using a rhodamine hydrazone immunosensor (see Figure 20) for separating *Mycobacterium avium* spp. *paratuberculosis* (MAP) bacteria from bovine feces, milk, and colostrum.

Fe nanoparticles with diethylene triamine pentaacetic acid (DTPA) or ethyl (dimethyl aminopropyl) carbodiimide (EDC)-*N*-hydroxy succinimide (NHS) as linkers were coupled with purified antibodies from hyperimmunized sera.

### 3.2. Hydrazone-Based Films and Coatings

In films and coatings, hydrazone groups are incorporated into the polymer matrix, either as pendant groups or as crosslinking agents. Upon exposure to UV or visible light, the double bond in the hydrazone group undergoes a photochemical reaction, leading to the formation of a new chemical bond and a change in the structure and properties of the material. This change can include a change in color, transparency, mechanical properties, or even chemical reactivity. For example, a photoresponsive coating containing hydrazone groups may change from opaque to transparent upon exposure to UV light or from soft to hard upon exposure to visible light. Hydrazone-based photoresponsive films and coatings have potential applications in a variety of fields, including sensors, smart windows, optical storage devices, and drug delivery systems [54,61]. They offer advantages such as high sensitivity, fast response times, and low toxicity, making them attractive for use in biomedicine and other sensitive applications [62].

**Figure 21:**
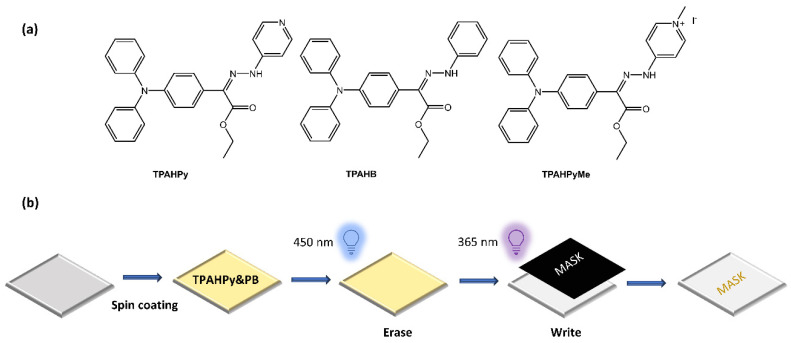
(**a**) Chemical structures of hydrazone-based AIE-active and photofluorochromic compounds and (**b**) fabrication of a rewritable photopattern based on TPAHPy/PB films [63].

Zhang et al. [63] have used photoswitchable hydrazone to develop a new photofluorochromic AIE system. They introduced luminogenic units with aggregation-induced emission (AIE) characteristics into the photoresponsive hydrazone to construct three AIE-active and photofluorochromic compounds, namely TPAHPy, TPAHB, and TPAHPyMe, incorporated into polybutadiene (PB) films to fabricate high-resolution, rewritable, and intensity-variable photopatterns (Figure 21).

TPAHPy was an excellent and reversible photoswitch with efficient fluorescence and fast photoisomerization. The TPAHPy/PB films were used to create detailed and quantitatively described images, which could be rewritten and had variable intensity.

Aprahamian and co-workers [64] reported a series of polyacrylate- and polymethacrylate-based polymer films having bistable hydrazone photoswitches as pendants. In their work, a correlation was observed between the *Z*/*E* isomer ratio and the T_g_ value of films: an increase in the amount of the *E* isomer in the polymer resulted in a noticeable rise in T_g_, which could reach up to 22 °C. They believed that this was due to the increased order induced by the hydrazones during the *Z*→*E* photoisomerization process, which led to a novel photo-hardening effect. Ma et al. [65] have developed two hydrazone-based photoswitches with excellent reversible photoisomerization properties and extremely long thermal stability. The authors have used this compound in the polymer matrix to fabricate transparent polymethylmethacrylate (PMMA) thin films and demonstrated that the excellent photochromic behavior could be further enhanced by coordinating them with various zinc salts. This coordination allowed for easy tuning of emission color during the intramolecular charge transfer (ICT) process. As a result of these properties, it was possible to create invisible, multicolor luminescent patterns using rewritable printing. The printed images had high resolution, excellent stability, and long retention times, making them ideal for anti-counterfeiting and ultrahigh density data storage applications.

## 4. Diarylethene Photoswitches

**Figure 22:**
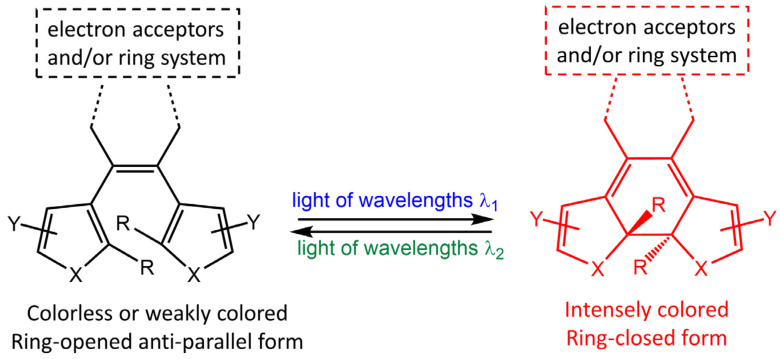
Chemical structures of open- and closed-ring isomers of diarylethene molecule and their properties.

Diarylethene molecules (DAE) are a relatively recent class of compounds with the unique property of changing color from colorless to red. They belong to the P-type photochromic molecules, which are photochemically reversible but thermally irreversible [66]. This means that once the photogenerated right-side isomers are formed, they are highly stable and do not easily revert to the left-side isomers in the dark at room temperature (Figure 22). Diarylethenes are derived from stilbene, featuring five-membered heterocyclic rings, such as thiophene or furan rings, in place of the phenyl rings found in stilbene. These modifications increase thermal stability for both the open and closed-ring isomers, making diarylethenes highly suitable for repeated cycles of coloration and discoloration [67,68].

Diarylethenes offer several benefits, including a high quantum yield and excellent thermal stability for both isomers. These qualities make them ideal for photoresponsive materials, as they exhibit strong performance. One area where they have been particularly useful is in supramolecular systems, where they can regulate photoresponsive assembly, induce morphological alterations, and trigger the gel-to-sol transition [54].

### 4.1. Diarylethene-Based Nanoparticles

**Figure 23:**
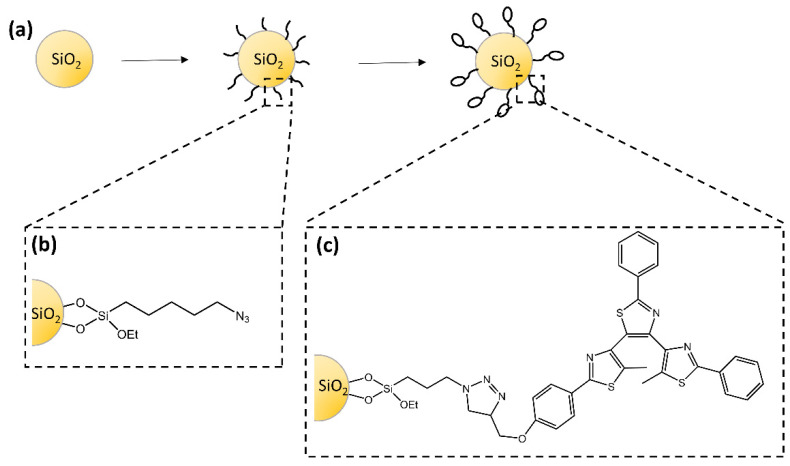
(**a**) Schematic steps to prepare photochromic silica nanoparticles with (**b**) azido-functionalized surface and (**c**) photochromic-functionalized surface [69].

The study of Dèbarre and co-workers [69] described the synthesis of silica nanoparticles functionalized with a diarylethene-based derivative that retained its photochromic and fluorescence properties (Figure 23). For the first time, fluorescence correlation experiments were used to observe the photoinduced ring-closure reaction. The authors suggested a model to explain the emission dynamics of the particles based on a photoinduced interconversion between the inactive and photoactive conformers.

**Figure 24:**
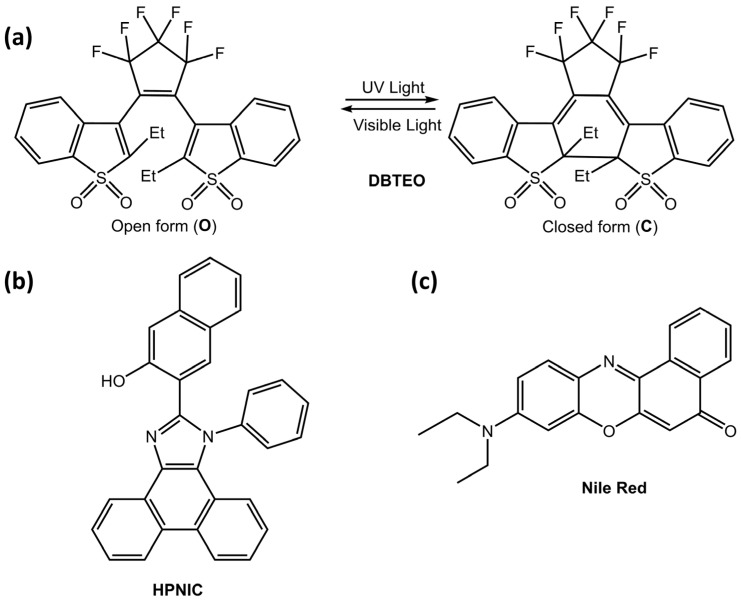
(**a**) Chemical structure and the photochromic reaction of DBTEO, (**b**) Chemical structure of HPNIC, (**c**) Chemical structure of Nile Red [70].

These particles could be helpful in imaging or bioimaging. Kim et al. [70] developed a new strategy for color-specific photoswitching, in which blue emission was selectively and completely switched, and orange emission was unaltered. The authors used a two-component system in polymer nanoparticles, composed of 3,3′-(perfluorocyclopent-1-ene-1,2-diyl)bis(2-ethylbenzo[b]thiophene 1,1-dioxide) (DBTEO) (Figure 24a) as a photoswitchable blue emitter and 3-(1-phenyl-1H-phenanthro[9,10-d]imidazol-2-yl)naphthalen-2-ol (HPNIC) (Figure 24b) or Nile Red (Figure 24c) as an orange emitter, respectively.

**Figure 25:**
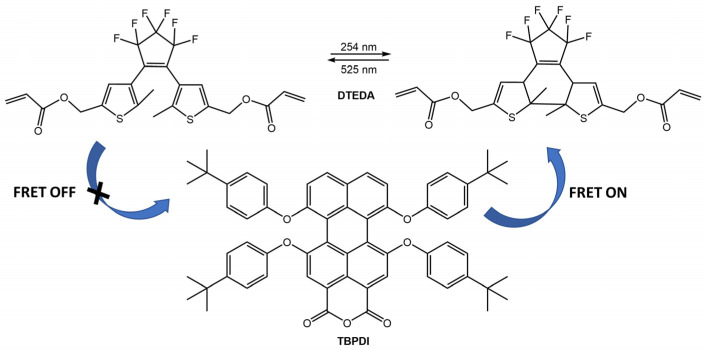
Chemical structures of TPFPNs components and representation of FRET process under UV/visible light irradiation [71].

Switching between UV and visible light, the system achieved 100% on/off blue emission of diarylethene, while the orange emission of the ESIPT dye remained unaltered. For the same bioimaging application, the new thermostable photoswitchable red fluorescent polymer nanoparticles (TPFPNs) were developed by Tian et al. [71]. The TPFPNs described in the article incorporate a red fluorescence dye, 1,6,7,12-tetra(*p*-*t*Bu-phenoxy)-3,4,9,10-di(anhydride) perylene (TBPDI), as the donor and a photochromic diarylethene derivative (DTEDA) as the acceptor (Figure 25). Upon exposure to UV light, DTEDA underwent a structural change from an opened-ring state (DTEDA-o) to a closed-ring form (DTEDA-c), which triggered a fluorescence resonance energy transfer (FRET) from TBPDI to DTEDA-c. This caused the red fluorescence to transition to a quenching state. In addition, TPFPNs were successfully used for reversible fluorescence bioimaging in Zebrafish.

The main feature of upconverting nanoparticles (UCNPs) is to efficiently absorb near-infrared light and transfer the upconverted excitation energy to activate appropriate photoswitches, such as diarylethene DAE, by UV or visible light. Lanthanide-doped upconverting nanoparticles (UCNPs) doped with either Er/Yb or Tm/Yb ion couples, specifically hexagonal-phase NaYF_4_ nanoparticles, are highly effective for sensitizing photochemical reactions. While most studies on these UCNPs have focused on their visible emissions, only a few have explored their UV upconversion emissions, due to the UV radiation is not feasible, such as in biological tissues and fluids [72,73].

### 4.2. Diarylethene-Based Films and Coatings

**Figure 26:**
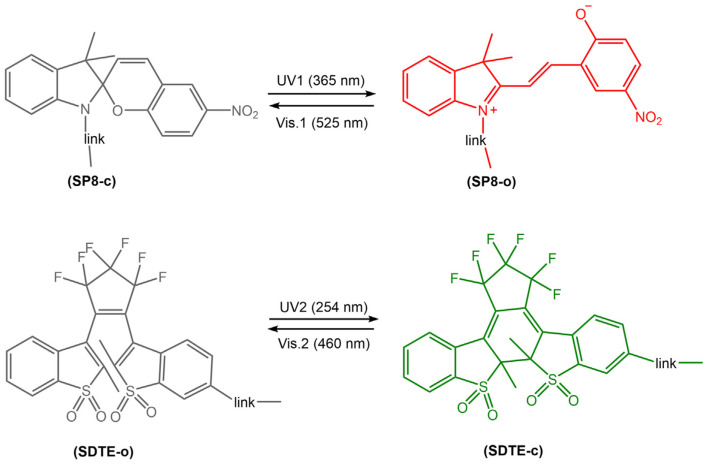
Chemical structure and photoisomerization of two photochromic fluorescent monomers to a photoswitchable multistate fluorescent polymer that can reversibly switch between multiple emission states (non-emission, red and green) [74].

Photoreactive diarylethene-based films and surfaces undergo a reversible photochemical reaction, changing their optical and surface properties upon exposure to light. The transition between these two isomers results in a change in the chemical and physical properties of the material, including color, fluorescence, wettability, adhesion, and mechanical properties. Thin films and surfaces made of diarylethene derivatives have been widely investigated for their applications in photonic devices, optical data storage, sensors, switches, and micro/nanoelectronics. These materials can be deposited onto various substrates, including glass, silicon, and polymers, using various techniques, such as spin coating, dip coating, and Langmuir-Blodgett deposition. The resulting films and surfaces exhibit excellent stability, high photoresponsiveness, and good reversibility, making them ideal candidates for various photo-actuated applications. Jiang et al. [74] have recently developed a new photoswitchable multilayer fluorescent polymer PMFP, obtained by copolymerization of butyl acrylate (BA) and styrene (St) with two fluorescent photochromic monomers, sulphonyl diarylethylene-linked 4-hydroxybutyl acrylate (SDTE) and spiropyran-linked methacrylate (SP8) (Figure 26).

**Figure 27:**
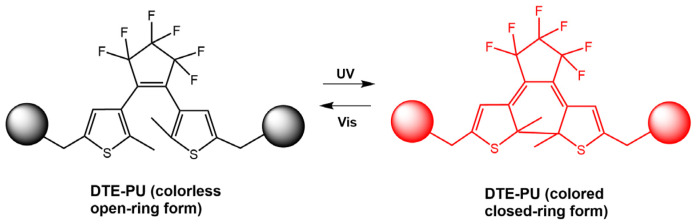
Chemical structures of PU (DTE–PU) film and its photo-switching behavior [75].

By changing the irradiation light, the ring-closed, non-fluorescent spiropyran units (SP8-c) and the ring-opened diarylethene units (SDTE-o) were converted to their corresponding fluorescent states. Furthermore, due to the FRET from the diarylethene core excited to the spiropyran, the emission of the polymer in the film was reversibly switched between non-emitting and emitting states in red or green (see Figure 26). The same authors [75] designed polymeric films based on photoswitchable polyurethanes via covalent bonding of photochromic diarylethene DTE-CH_2_OH (Figure 27). Upon irradiation with UV light, the colorless DTE–PU film changed to red and could be reversibly switched to its original state via illumination with visible light. The DTE-PU films exhibited rapid and reversible isomerization and more than 20-fold photo reversibility, as well as long-term optical stability. In addition, photowritten patterns on the films remained stable for over two weeks. This made the films promising candidates for various applications, such as optical archiving.

**Figure 28:**
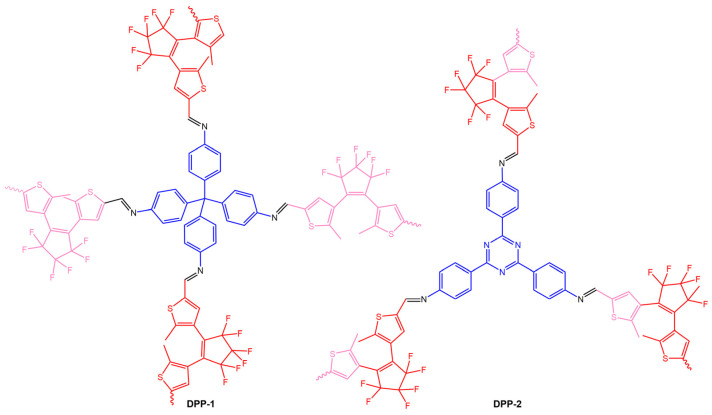
Chemical structures of DPP-1 and DPP-2 obtained [76].

The work of Fu et al. [76] reported two diarylethene-based conjugated polymer networks DPP-1 and DPP-2 (Figure 28), obtained by Schiff-base polymerization of the photochromic unit, 1,2-bis(5-formyl-2-methylthiophen-3-yl)perfluorocyclopentene (DEA-CHO), and conjugated polyamine tetra(*p*-aminophenyl)methane (TAPM) or 1,3,5-tris(4-aminophenyl)triazine (TAPT).

The authors observed an ultrafast photochromic transition between the yellow open form and green closed form of DPP-1 and DPP-2 through alternating UV and visible irradiation. Additionally, they created DPP-1/PMMA and DPP-2/PMMA films by incorporating diarylethene-based conjugated polymer networks into a PMMA matrix, which also exhibited fatigue resistance and photochromic reversibility.

### 4.3. Diarylethene-Based MOFs

**Figure 29:**
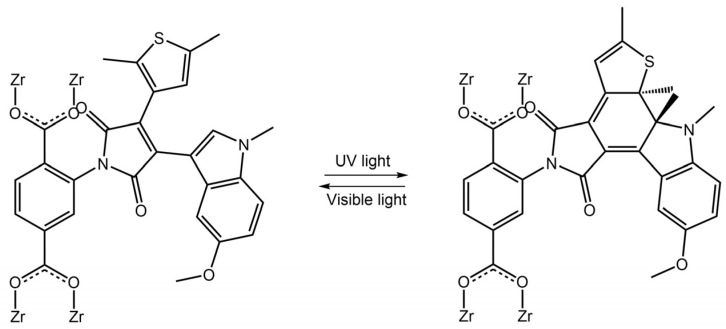
Scheme of DAE transformations inside DAE-UiO-66 pores under UV-light and visible light [80].

Photoresponsive diarylethene-based metal-organic frameworks (MOFs) show advantageous properties and numerous potential practical applications. Due to the intrinsic porosity that characterizes metal-organic-frameworks, most highlighted examples can be applied in selective adsorption or controlled cargo release and data storage and optoelectronics [77,78,79]. Butova and co-workers [80] reported a new photosensitive MOF, modified UiO-66-NH_2_, one of the most popular MOFs, with diarylethene molecules (DAE, 4-(5-methoxy-1,2-dimethyl-1H-indol-3-yl)-3-(2,5-dimethylthiophen-3-yl)-4-furan-2,5-dione), obtaining a highly crystalline porous compound (Figure 29).

**Figure 30:**
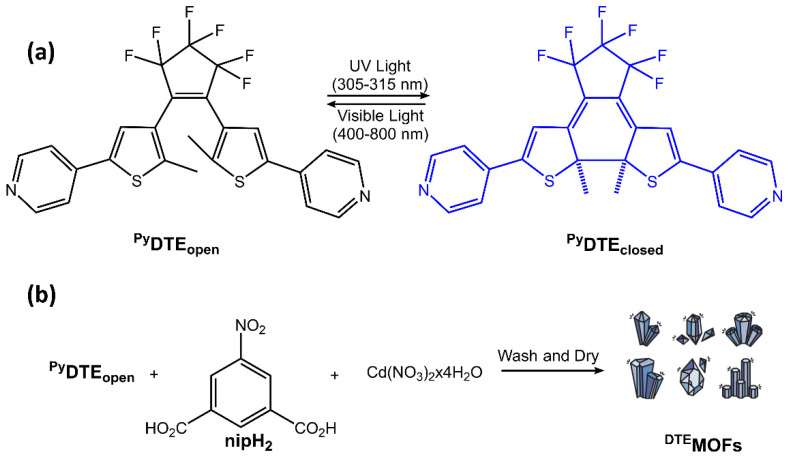
(**a**) Reversible photochemical cyclization of PyDTEopen upon UV and visible light irradiation. (**b**) A typical procedure for the synthesis of ^DTE^MOF [81].

The DAE molecules inside UiO-66-NH_2_ pores had an open conformation. With visible light irradiation at 450 nm, the DAE molecules changed their conformation in closed form. The authors demonstrated that this transformation could stimulate the hydrogen adsorption-desorption process. Visible light irradiation increased the H_2_ capacity of modified MOF, while UV light decreased it. Diarylethene molecules were also used in Sato et al. work [81] to develop a photochemically crushable and regenerative metal-organic framework (^DTE^MOF) by a combination of a photochromic ligand ^Py^DTE_open_ and 5-nitro isophthalate (nip^2−^) with Cd^2+^, reported in Figure 30:

The obtained ^DTE^MOF colorless crystals could accommodate guest molecules in its pores, and when ^DTE^MOF was exposed to UV light, its crystalline structure was converted into a homogeneous, blue-colored solution thanks to ring-closing isomerization of photochromic DTE. After exposure to visible light, the colorless MOF was regenerated. Li et al. [82] incorporated photochromic DAE into lanthanide MOF, and they chose Eu^3+^ ions to have a luminescent host MOF. The emission band of the ion overlapped with the absorption spectra of closed-form DAE but did not with the absorption spectra of open-form DAE. Therefore, reversible switching of DAE between its two forms could provide remote control of the luminescence of its host MOF. When exposed to UV radiation at 300 nm, the DAE component within the MOF underwent photocyclization, causing the powder’s color to shift from white to blue. Repeated exposure to UV and visible light 20 times resulted in a slight decrease in the photoluminescence intensity, demonstrating fatigue resistance. MOFs are also emerging as an essential class of biomedical nanomaterials due to their porosity, biocompatibility, synthetic tunability, and structural regularity. MOFs can integrate nanoparticles and/or biomolecules. More importantly, the stimulus-responsive release offers an excellent opportunity for precise imaging-guided tumor treatment for all-in-one theranostics [83,84].

## 5. Spiropyran Photoswitches

**Figure 31:**
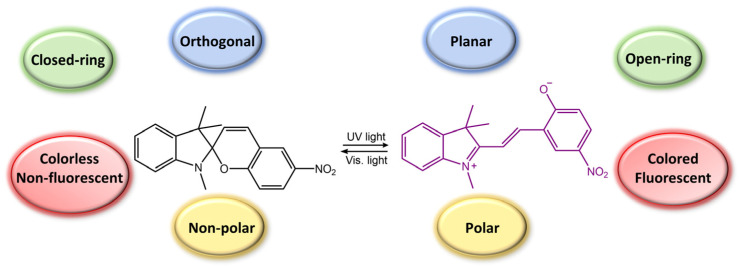
Spiropyran photoisomerization process and isomers characteristics.

Spiropyran is a photochromic molecule that exhibits reversible changes in color or optical properties upon exposure to light of specific wavelengths. Spiropyran molecules typically exist in two forms: a colorless, non-planar, closed-ring form and a colored, planar, open-ring form. When exposed to ultraviolet (UV) light or visible light of a specific wavelength, spiropyran molecules undergo a photoisomerization reaction, transforming the non-planar, colorless form into the planar, colored form (Figure 31) [85]. The photoisomerization process involves breaking a carbon-oxygen bond in the spiro ring and forming a new carbon-carbon bond. This leads to a significant change in molecular geometry and electronic structure. The colored form of spiropyran (SP) is called merocyanine (MC), which absorbs light in the visible region and has a distinct color depending on the substituents on the molecule. Upon exposure to light of a different wavelength, the merocyanine form can undergo a reverse reaction, or thermal relaxation, to convert back to the spiropyran form [2,8].

The photoresponsive effect of spiropyran has numerous potential applications in areas such as photochromic materials, optical storage, photonic devices, sensors, and actuators. For example, spiropyran-based materials can be used as reversible optical switches or sensors, as the color change can be used to indicate the presence or absence of a particular stimulus. In addition, spiropyran can be incorporated into polymers, films, or surfaces to create photoresponsive coatings that can be tuned to respond to specific wavelengths of light [86]. Spiropyran-based compounds have been studied extensively for their potential use in biomaterials due to their reversible photochromic properties, biocompatibility, and ease of functionalization [87,88].

### 5.1. Spiropyran-Based Hydrogels and Particles

Hydrogels have a high-water content and share similarities with the extracellular matrix, allowing them to potentially function in aqueous environments and exchange fluids. A recent work by Aggarwal and co-workers [89] reported a new hydrogel based on the concept of origami that could mimic the complex functions of living organisms. Their photoactive hydrogel displayed various dynamic shape changes using a single material upon irradiation with local light. The hydrogel was created using spiropyran photoswitches with *N*-isopropylacrylamide (NIPAM) monomer and *N*,*N*′-methylenebisacrylamide (MBAAm) crosslinker. The hydrogel sheet demonstrated 83% contraction of the original size upon irradiation and recovered the original swelling state in the dark. Furthermore, this contraction-expansion process was found to be highly reversible by alternately switching the light on and off.

**Figure 32:**
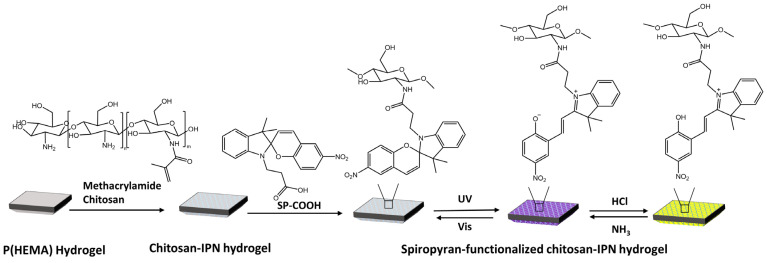
Preparation of SP-functionalized chitosan-IPN hydrogels and the structural conversion of SP with light and gases [90].

Vales et al. [90] have developed light- and acid/base-reactive hydrogels based on spiropyran-modified poly(hydroxyethyl methacrylate) p(HEMA) with methacrylamide-chitosan (Figure 32).

When exposed to UV light, SP changes into MC. Treating MC with HCl vapor resulted in the protonation of MC, turning it into MCH^+^ and causing the hydrogel to change from purple to yellow. To retrieve the MC isomer, MCH^+^ was deprotonated by treating it with NH_3_ vapor.

**Figure 33:**
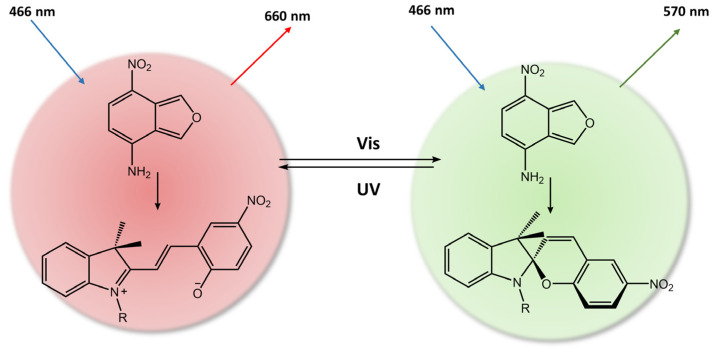
Photoresponsive dual-color fluorescent PVA hydrogel based on FRET [91].

In the work of Zhang et al. [91], a hydrogel made of poly(vinyl alcohol) (PVA) was created with unique fluorescence properties by incorporating dual fluorescent nanoparticles (DCFNs) composed of spiropyran-modified β-cyclodextrin (β-CD-SP) and nitrobenzoxadiazolyl (NBD) derivates. The SP portion of the nanoparticles functioned as an acceptor, which enabled the photoisomerization process to either quench or recover the fluorescence of the NBD portion, utilizing the fluorescence resonance energy transfer (FRET) effect (see Figure 33).

**Figure 34:**
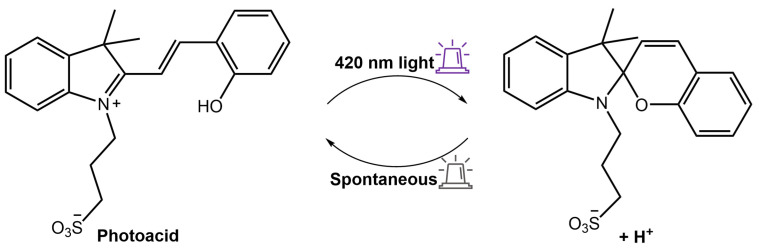
Scheme of the spiropyran-based photoacid that reversibly releases and captures H^+^ in response to 420 nm light [92].

As a result, when exposed to ultraviolet or visible light, the hydrogel emitted a red fluorescence peak of merocyanine (MC) at 660 nm or a green fluorescence peak of NBD at 550 nm, respectively. This distinctive property made the composite hydrogel suitable for various applications such as sensors, optical switches, and biomimetic components. Furthermore, Zhang et al. [92] used a spiropyran-based photoacid to trigger the phase transition of PNIPAMm using light. The hydrogel system comprised a copolymer based on poly(*N*-isopropylacrylamide-co-acrylic acid) (PNIPAm-co-PAA) and spiropyran that could reversibly release and capture H^+^ through a high-efficiency light-switchable isomerization (Figure 34).

PNIPAm-co-PAA exhibited a lower critical solution temperature LCST pH-dependent. The LCST can be increased or decreased by the release and capture of H^+^ by the photoacid, resulting in a fast (<0.5 min) and reversible phase transition. Amer et al. [93] have used spiropyran-conjugated *N*-isopropylacrylamide (NIPPAM) to create a photoresponsive hydrogel that could self-adhere to the application site upon swelling and deswelling for easy removal when illuminated with light.

**Figure 35:**
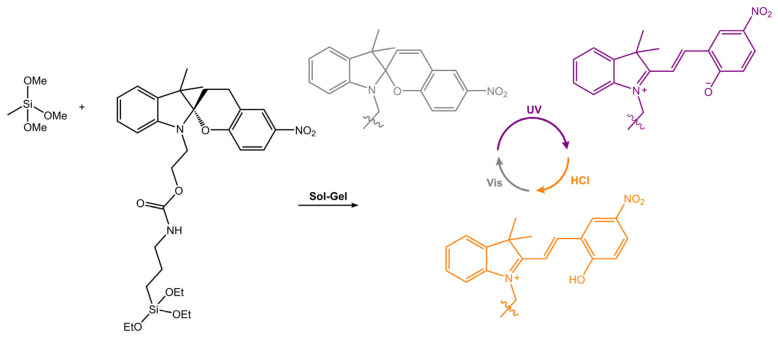
Images of SP10-MTMS before (SP) and after UV irradiation (MC) and after treating the irradiated sample with a 1 M HCl solution (MCH^+^) [94].

Micro- and nanoparticles that can change their properties, such as wettability and polarity, reversibly have garnered significant attention due to their potential use in the smart materials field. In a recent work by Feinle and co-workers [94], the silylated spiropyran derivative was covalently attached into porous monolithic silsesquioxane frameworks (Figure 35). The surface showed a dependence between the predominant isomer and the surface polarity and water wettability. Indeed, the contact angle of a drop of water on the surface varied from 146° to 100° by irradiation with UV light.

**Figure 36:**
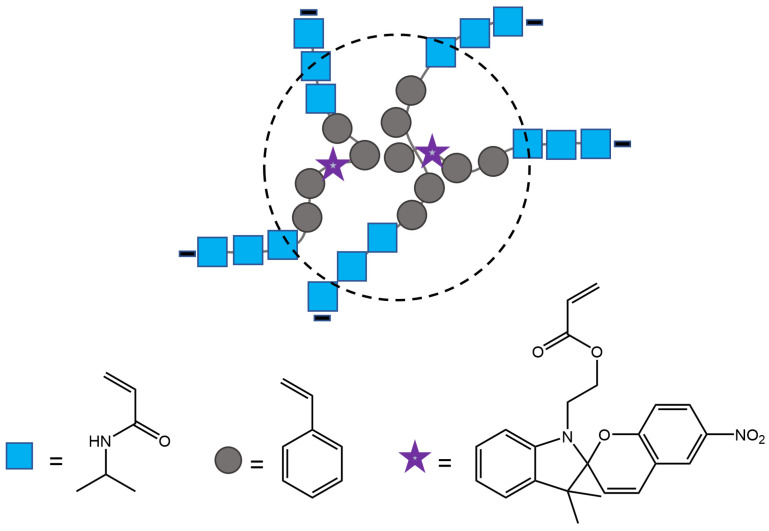
Schematic representation of polymer nanoparticle and its components [95].

Zhu et al. [95] have synthesized polymer nanoparticles with 40−400 nm diameter, capable of reversible fluorescence photoswitching with alternating UV and visible light that can have potential utility as sensitive displays and biological markers. The nanoparticles consisted of primary monomeric units of isopropylacrylamide (NIPAM) and styrene, small amounts of divinylbenzene (DVB) as a crosslinker, and 5-(1,3-dihydro-3,3-dimethyl-6-nitrospiro[2H-1-benzopyran-2,2′-(2H)-indole]) ethylacrylate (SP) as a photoreactive unit. Small amounts of butyl acrylate (BA) were added to the nanoparticles to lower their glass transition temperature (T_g_). (Figure 36). Also, the immobilization of dye inside hydrophobic pockets of nanoparticles improved its photostability, rendering it more resistant than the same dyes in the solution.

### 5.2. Spiropyran-Based Films and Coatings

**Figure 37:**
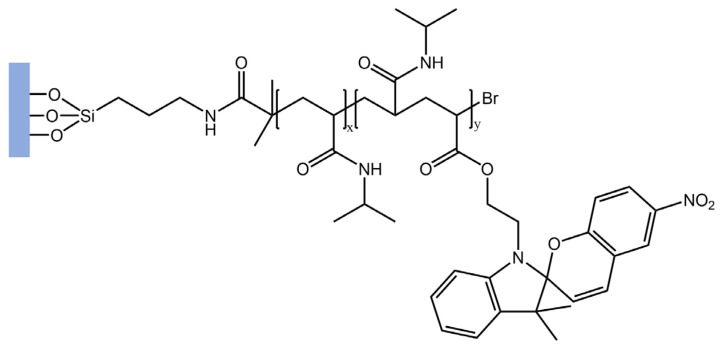
Schematic representation of PNIPAAm-b-P(NIPAAm-co-SP) brushes [96].

In a recent study [96], the authors used surface-initiated atom transfer radical polymerization (SI-ATRP) to tether poly(*N*-isopropylacrylamide)-spiropyran (PNIPAAm-SP) copolymers onto glass substrates that were previously activated using ultra-violet ozone (UVO) irradiation. A PNIPAAm block was grafted onto the glass substrates, followed by a second block containing mixed NIPAAm and spiropyran acrylate (SPA) to build PNIPAAm-b-P(NIPAAm-co-SP) brushes (Figure 37).

**Figure 38:**
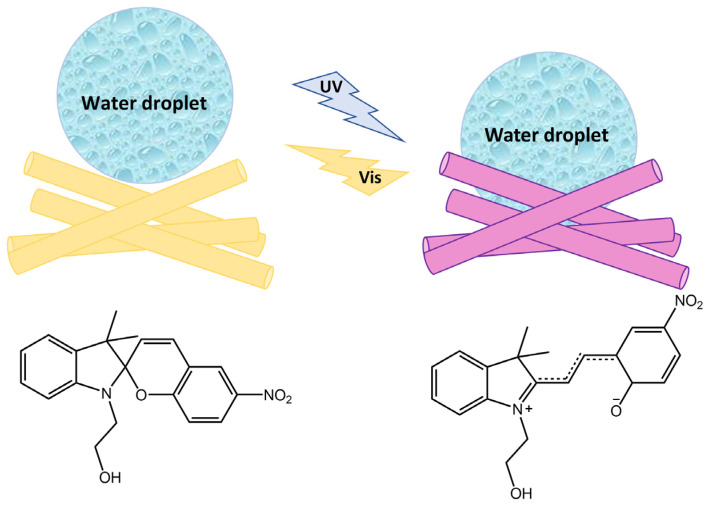
Schematic illustration of the photoresponsive wettability transition of fiber materials by SP-OH photoisomerization [97].

The effects of UVO irradiation time and spiropyran-containing block polymerization time on cell sheet formation and detachment characteristics were evaluated. The photoresponsiveness of SPA was analyzed using UV-visible spectroscopy, while the presence and thickness of the grafted polymer layers were confirmed. The authors found that L929 mouse fibroblast cells successfully formed a complete cell sheet, which could be detached in a trypsin-free procedure by applying temperature or light stimuli. In a study by Wang [97], novel fiber materials were developed by mixing *N*-hydroxyethyl spiropyran (SP-OH) and polyacrylonitrile (PAN) through physical doping and electrospinning (Figure 38).

**Figure 39:**
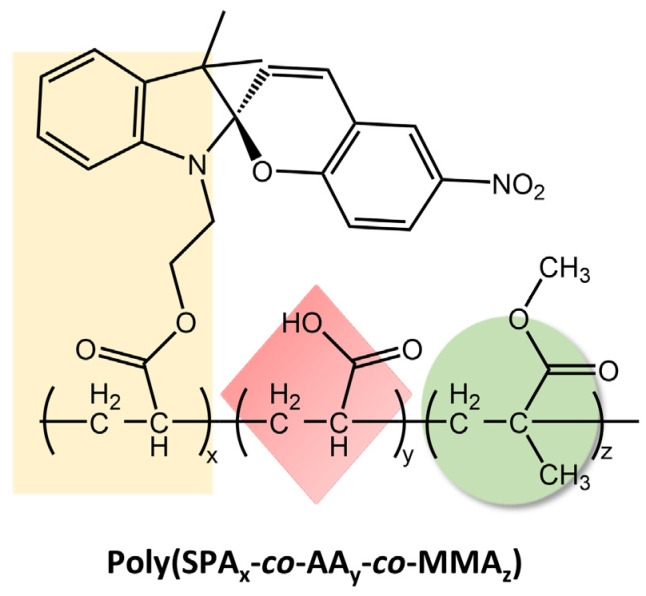
Chemical structure of reversible photoresponsive poly(SPAx-co-AAy-co-MMAz) material [99].

These materials reversibly changed their wettability and humidity in response to ultraviolet-visible (UV-Vis) light irradiation due to the photoisomerization mechanism of the spiropyran chromophore. When exposed to UV light, the SP-OH molecules adopted a colored polar open-ring state, resulting in electrostatic attraction with water. However, they became colorless and non-polar when exposed to visible light, losing their attraction effect. By repeatedly switching between these states, the wettability and ambient humidity of the electro-spun films could be controlled. The tensile strength and the extent of reversible changes in wettability and humidity under UV-Vis irradiation depended on the amount of SP-OH added. Karimipour et al. [98] also utilized hydroxyl spiropyran (SP-OH) for designing and preparing hydrochromic and photoresponsive polymers based on poly(methyl methacrylate-co-hydroxyethyl methacrylate) (P(MMA-co-HEMA)) and poly(methyl methacrylate-co-butyl acrylate) (P(MMA-co-BA)), with SP-OH as a dopant. Subsequently, the copolymers solutions with photoactive properties were utilized for the fabrication of either nano-fibers through electrospinning (MH/SP@NF and MB/SP@NF) or films via drop-casting (MH/SP@F and MB/SP@F) in order to explore their functionalities. The fibers and the films demonstrated hydrochromic and humidity-responsive behavior together with photoresponsivity, combined with improved reversibility and reusability. In the same way, Li et al. [99] have prepared electrospun films with reversible photoresponsive wettability and the ability to regulate microenvironmental relative humidity developed from acryloyl-spiropyran (SPA), acrylic acid (AA), and methyl methacrylate (MMA) (poly(SPA_x_-co-AA_y_-co-MMA_z_)), represented in Figure 39.

The wettability was changed by exposing them to alternating UV and visible light due to the reversible transition of spiropyran. Furthermore, the presence of acrylic acid in the polymers caused a controllable “double hydrogen bonds” synergistic effect with phenol-oxygen bonds from spiropyran units, which increased the range over which microenvironmental relative humidity was regulated. The amount of acrylic acid used determined the color, wettability change, and the reversible variation of microenvironmental relative humidity regulation under UV/visible irradiation. The same authors have constructed a photoresponsive material that could reversibly regulate the humidity of the microenvironment [100]. The material was made by a synergistic layer (amino groups) and a regulating layer (spiropyran compounds) on the surface of polyacrylonitrile (PAN) electrospun membranes.

The wettability and microenvironment humidity could be controlled by light due to spiropyran transformation. Moreover, the amino groups on the surface assisted the ring-opened merocyanine in absorbing water molecules, thus playing a synergistic role.

**Figure 40:**
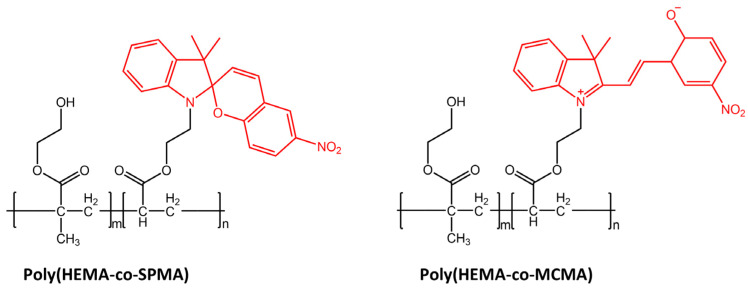
Chemical structures of hydrophobic poly(HEMA-co-SPMA) and hydrophilic poly(HEMA-co-MCMA) used by Wang et al. in their new photoresponsive spiropyran-coated nanostructured surface [102].

Light was also used for cell manipulation (e.g., cell release) due to its high spatiotemporal precision and non-invasion [101]. Li et al. [102] in their study have designed a new photoresponsive spiropyran-coated nanostructured surface using hydrophobic poly(HEMA-co-SPMA) and hydrophilic poly(HEMA-co-MCMA) (Figure 40).

This new surface showed reversible capture and release of targeted cells mediated by photo-triggered wettability transition.

Qing Xu and co-workers [103] used spiropyran molecule in a photoresponsive controlled pesticide release film system. They used PEG-supported spiropyran as a carrier and chitosan as a film-forming additive. Acetamiprid, a new broad-spectrum insecticide with acaricidal activity, was encapsulated in the photoresponsive film, and the authors observed his controlled release from the biodegradable photosensitive film.

### 5.3. Spiropyran-Based MOFs

In Garg’s work [104], the spiropyran molecule was used to photo-regulate the conduction of the metal-organic framework (MOF) reversibly. The authors reported the design and incorporation of spiropyran into the relatively hydrophobic pores of UiO-67-MOF-films (SP@UiO-67-MOF). The increased electronic conductance in the MOF was attributed to changes in its microstructure following the isomerization process. This led to an increase in ionization potential, longer molecular length, and a more extensive conjugated π-electron system. In another work, Heinke and co-workers [105] anchored spiropyran into the pores of a MOF, Cu_2_(e-BPDC)_2_(dabco). They studied the conductivity of guest molecules (D_2_O) within the material, both in its neat form and after water loading. They found that the neat Cu_2_(SP-BPDC)_2_(dabco) had very low conductivity, but the addition of water increased the conductivity significantly. They also found that UV irradiation could modulate the proton conduction of the guest molecules by switching spiropyran to its more polar isomer, merocyanine. This effect was due to the strong bonding of merocyanine with the guest molecules, which suppressed proton conduction. Chen and colleagues investigated the photo modulation of proton conduction in spirogyra-encapsulated MOFs [106].

**Figure 41:**
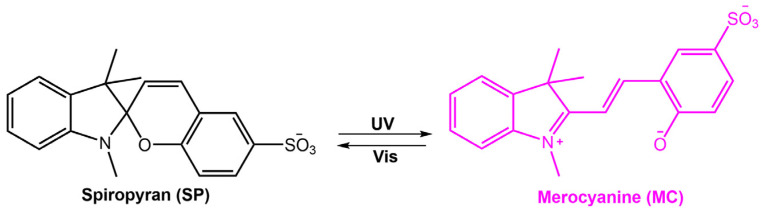
Chemical structures and photoisomerization process of SP and MC reported in [106].

They encapsulated sulfonated spiropyran (SSP) into ZIF-8 cavities to create SSP@ZIF-8 hybrid membranes (see Figure 41). SSP@ZIF-8 containing 10 wt.% of SPP showed the highest proton conductivity. The phenolate moiety of merocyanine and sulfonate groups in the ZIF-8 membrane facilitated proton conduction through the formation of a hydrogen-bond network with water molecules inside the cavities. The high proton conductivity decreased after being exposed to visible light and undergoing the merocyanine to spiropyran transformation. The resulting hybrid membrane was able to switch its proton conductivity on/off depending on the light.

**Figure 42:**
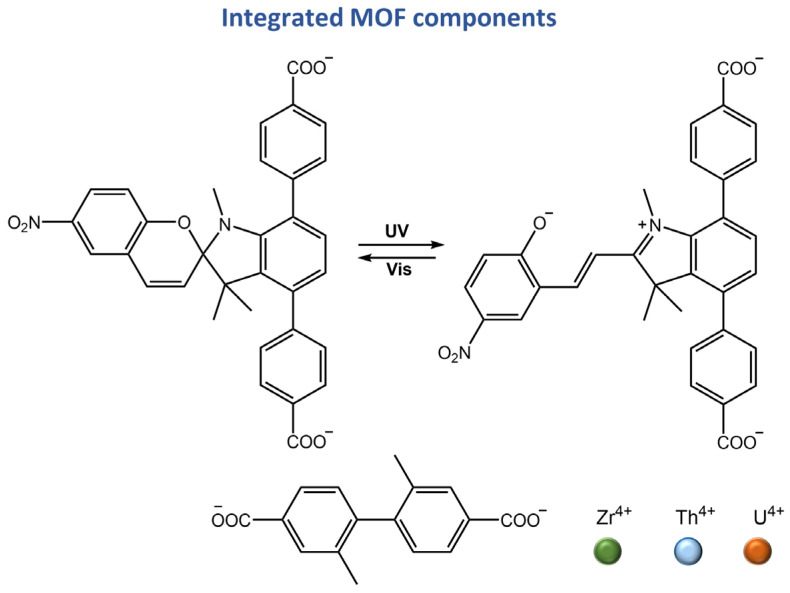
Chemical structures of MOFs components reported in [107].

In a recent work by Martin et al. [107], a correlation between photophysics and electronics was established for actinide-containing metal-organic frameworks (An-MOFs) based on the excitation wavelength. A stepwise approach for dynamically modulating electronic properties was applied to actinide-based heterometallic MOFs by integrating photochromic linkers, spiropyran-based linker, 4,4′-(1′,3′,3′-trimethyl-6-nitrospiro[chromene-2,2′-indoline]-4′,7′-diyl) dibenzoic acid (H_2_TNDA) (Figure 42).

Different methods, such as optical cycling, modeling of the density of states, conductivity measurements, and photoisomerization kinetics, have been used to understand the process of tailoring optoelectronic properties of An-MOFs. Additionally, the first photochromic MOF-based field-effect transistor was constructed, which could change the field-effect response through light exposure. In a recent study, Yang and colleagues [108] utilized a combination of lanthanide MOF and spiropyran photochromic molecules to develop intelligent fluorescent materials that have the potential for dynamic anticounterfeiting applications. This approach offered several advantages, such as the ability to create reversible anticounterfeiting patterns. Additionally, it displayed both fluorescence and color changes with UV light exposure.

**Figure 43:**
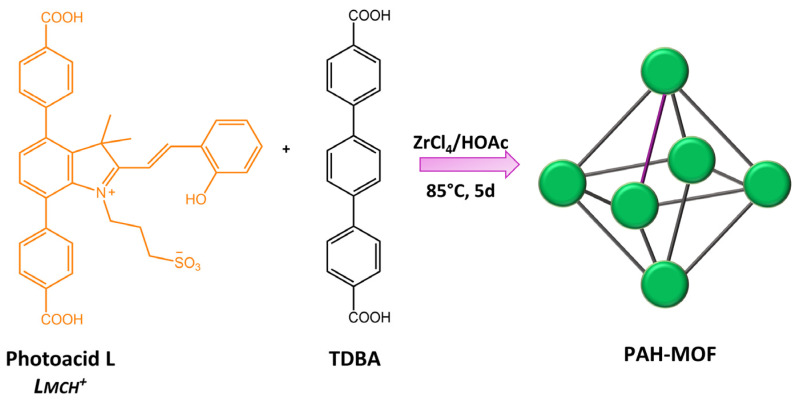
Schematic illustration of the synthesis of PAH-MOF using a mixed linker approach [109].

Zhang and co-workers [109] incorporated a photoacid metastable state, protonated-merocyanine, into zirconium(IV)-based UiO-topological metal-organic framework (MOF) material (form PAH-MOF) (Figure 43).

Although the PAH-MOF did not exhibit photoactivity in its solid state, it demonstrated remarkable photochromism when suspended in acidified water and ethanol under visible light. The observed color change was accompanied by a significant alteration in fluorescence, indicating the possibility of using this material for anti-counterfeiting purposes. In addition, this study contributed to a better comprehension of the influence of MOFs on spiropyran derivative isomerization.

## 6. Comparison among the Different Systems

Summarizing the data collected in this review on photoresponsive biomaterials based on azobenzenes, diarylethenes, hydrazones, and spiropyrans, we can briefly compare the different systems. Azobenzene-based hydrogels have shown promise in several biomedical applications due to their ability to regulate the gel’s mechanical properties through photoisomerization. Hydrazone-based hydrogels offer reversible changes in mechanical properties through light-induced crosslinking or de-crosslinking. Spiropyran-based hydrogels have unique fluorescence properties and can self-adhere to the application site upon swelling. Each type of photochromic hydrogel has advantages and can be tailored to specific applications. Micro- and nanoparticles functionalized with photochromic compounds offer potential utility as sensitive displays, biological markers, and smart materials due to their ability to switch properties upon light irradiation.

Azobenzene-based MOFs are often used for gas separation and dye adsorption, diarylethene-based MOFs are usually applied in hydrogen storage and cargo release, and spiropyran-based MOFs are often used for proton conduction and electronic modulation. Additionally, the type of functional group incorporated into the MOF structure affects the specific properties and applications of the resulting material. For example, incorporating sulfonate or phenolate groups in spiropyran-based MOFs enhances the proton conductivity, while introducing amines in diarylethene-based MOFs promotes CO_2_ adsorption.

Overall, films and coatings based on azobenzenes, hydrazones, diarylethanes, and spiropyrans offer unique opportunities for developing photoresponsive devices and smart materials. These compounds share many common properties and applications but exhibit distinct differences in photoisomerization behavior, chemical stability, and spectral properties.

## 7. Conclusions

Photoresponsive biomaterials have emerged as a promising area of research due to their ability to regulate biological interactions and cellular behaviors in response to light. This article provided an overview of photoresponsive biomaterials’ design, synthesis, and applications, including photochromic molecules, photocleavable linkers, and photoreactive polymers. Specific examples of azobenzene, hydrazones, diarylethene, and spiropyran-based materials, like hydrogels and metal-organic frameworks, were discussed, highlighting their potential for applications such as antibacterial coatings, drug delivery, and optical sensing. The various approaches used to control the photoresponsive behavior of these materials were also highlighted, along with their potential applications in drug delivery, tissue engineering, and biosensing. Overall, the development of photoresponsive biomaterials holds great promise for advancing biomedical research and developing advanced biomaterials with enhanced functionality.

## Data Availability

Not applicable.
